# ECDC’s latest publications

**DOI:** 10.2807/1560-7917.ES.2019.24.26.1906272

**Published:** 2019-06-27

**Authors:** Eurosurveillance editorial team

**Affiliations:** 1 European Centre for Disease Prevention and Control (ECDC), Stockholm, Sweden

**Keywords:** infectious diseases, public health, Europe

 

**Rapid risk and outbreak assessments**

 

Dengue outbreak in Réunion, France, and associated risk of autochthonous outbreak in the EU/EEA

Date of publication: 18 June 2019

https://ecdc.europa.eu/en/publications-data/rapid-risk-assessment-dengue-outbreak-reunion-france-and-associated-risk

**

*Chlamydia trachomatis* false-negative test results by Aptima Combo 2 CT/NG assay (Hologic) in the EU/EEA, 2019

Date of publication: 17 June 2019

https://ecdc.europa.eu/en/publications-data/rapid-risk-assessment-chlamydia-trachomatis-false-negative-test-results-aptima

 

Multi-country outbreak of *Listeria monocytogenes* clonal complex 8 infections linked to consumption of cold-smoked fish products

Date of publication: 4 June 2019

https://ecdc.europa.eu/en/publications-data/multi-country-outbreak-listeria-monocytogenes-fish-products

 

Regional outbreak of New Delhi metallo-betalactamase-producing carbapenem-resistant Enterobacteriaceae, Italy, 2018–2019

Date of publication: 4 June 2019

https://ecdc.europa.eu/en/publications-data/RRA-new-delhi-metallo-beta-lactamase-producing-CRE

 

Who is at risk of measles in the EU/EEA?

Date of publication: 28 May 2019

https://ecdc.europa.eu/en/publications-data/risk-assessment-measles-eu-eea-2019

 

Ebola virus disease outbreak in North Kivu and Ituri Provinces, Democratic Republic of the Congo – fourth update

Date of publication: 17 April 2019

https://ecdc.europa.eu/en/publications-data/rapid-risk-assessment-ebola-virus-disease-outbreak-north-kivu-and-ituri-0

 

Zika virus transmission worldwide

Date of publication: 11 April 2019

https://www.ecdc.europa.eu/en/publications-data/zika-virus-transmission-worldwide

 

Cyclone Idai - risk of communicable diseases in southern Africa

Date of publication: 11 April 2019

https://www.ecdc.europa.eu/en/publications-data/rapid-risk-assessment-cyclone-idai-risk-communicable-diseases-southern-africa

 

**Technical reports**

 

Collection and analysis of whole genome sequencing data from food-borne pathogens and other relevant microorganisms isolated from human, animal, food, feed and food/feed environmental samples in the joint ECDC–EFSA molecular typing database

Date of publication: 29 May 2019

https://ecdc.europa.eu/en/publications-data/collection-and-analysis-whole-genome-sequencing-data-food-borne-pathogens-and

 

Third external quality assessment on species identification and antimicrobial susceptibility testing of Campylobacter, 2017

Date of publication: 28 May 2019

https://ecdc.europa.eu/en/publications-data/third-external-quality-assessment-species-identification-and-antimicrobial

 

Developing a reporting system for the surveillance of HIV drug resistance in Europe

Date of publication: 23 May 2019

https://ecdc.europa.eu/en/publications-data/developing-reporting-system-surveillance-hiv-drug-resistance-europe

 

Proficiency test for *Listeria monocytogenes* whole genome assembly 2018

Date of publication: 16 May 2019

https://ecdc.europa.eu/en/publications-data/proficiency-test-listeria-monocytogenes-whole-genome-assembly-2018

 

ECDC strategic framework for the integration of molecular and genomic typing into European surveillance and multi-country outbreak investigations

Date of publication: 4 April 2019

https://ecdc.europa.eu/en/publications-data/ecdc-strategic-framework-integration-molecular-and-genomic-typing-european

 

**Surveillance reports**

 

Monthly measles and rubella monitoring report, June 2019

Date of publication: 14 June 2019

https://ecdc.europa.eu/en/publications-data/monthly-measles-and-rubella-monitoring-report-june-2019

 

Influenza virus characterisation, May 2019

Date of publication: 10 June 2019

https://ecdc.europa.eu/en/publications-data/influenza-virus-characterisation-may-2019

 

Influenza virus characterisation, Summary Europe, April 2019

Date of publication: 15 May 2019

https://ecdc.europa.eu/en/publications-data/influenza-virus-characterisation-summary-europe-april-2019

 

Monthly measles and rubella monitoring report, May 2019

Date of publication: 10 May 2019

https://ecdc.europa.eu/en/publications-data/monthly-measles-and-rubella-monitoring-report-may-2019

 

Influenza virus characterisation, March 2019

Date of publication: 24 April 2019

https://ecdc.europa.eu/en/publications-data/influenza-virus-characterisation-march-2019

 

Monthly measles and rubella monitoring report, April 2019

Date of publication: 12 April 2019

https://ecdc.europa.eu/en/publications-data/monthly-measles-and-rubella-monitoring-report-april-2019

 

**Annual Epidemiological Report series on communicable diseases in Europe**

****

**Published in June:**

****

Chapter on diphtheria for 2017

https://ecdc.europa.eu/en/publications-data/diphtheria-annual-epidemiological-report-2017

 

Chapter on congenital toxoplasmosis for 2016

https://ecdc.europa.eu/en/publications-data/congenital-toxoplasmosis-annual-epidemiological-report-2016

 

Chapter on brucellosis for 2017

https://ecdc.europa.eu/en/publications-data/brucellosis-annual-epidemiological-report-2017

 

Chapter on hepatitis B for 2017

https://ecdc.europa.eu/en/publications-data/hepatitis-b-annual-epidemiological-report-2017

 

Chapter on smallpox for 2017

https://ecdc.europa.eu/en/publications-data/smallpox-annual-epidemiological-report-2017

 

Chapter on giardiasis (lambliasis) for 2017

https://ecdc.europa.eu/en/publications-data/giardiasis-lambliasis-annual-epidemiological-report-2017

 

Chapter on Lassa fever for 2017

https://ecdc.europa.eu/en/publications-data/lassa-fever-annual-epidemiological-report-2017

 

Chapter on Q fever for 2017

https://ecdc.europa.eu/en/publications-data/q-fever-annual-epidemiological-report-2017

 

Chapter on West Nile virus infection for 2017

https://ecdc.europa.eu/en/publications-data/west-nile-virus-infection-annual-epidemiological-report-2017

 

Chapter on yellow fever for 2017

https://ecdc.europa.eu/en/publications-data/yellow-fever-annual-epidemiological-report-2017

****

**Published in May:**

 

Chapter on invasive pneumococcal disease for 2017

https://ecdc.europa.eu/en/publications-data/invasive-pneumococcal-disease-annual-epidemiological-report-2017

 

Chapter on poliomyelitis for 2017

https://ecdc.europa.eu/en/publications-data/poliomyelitis-annual-epidemiological-report-2017

 

Chapter on Crimean-Congo haemorrhagic fever for 2017

https://www.ecdc.europa.eu/en/publications-data/crimean-congo-haemorrhagic-fever-cchf-annual-epidemiological-report-2017

 

Chapter on zoonotic influenza for 2018 

https://ecdc.europa.eu/en/publications-data/zoonotic-influenza-annual-epidemiological-report-2018

 

**Published in April:**

****

Chapter on Chikungunya virus disease for 2017

https://ecdc.europa.eu/en/publications-data/chikungunya-virus-disease-annual-epidemiological-report-2017

 

Chapter on Shiga toxin/verocytotoxin-producing Escherichia coli (STEC/VTEC) infection for 2017 

https://ecdc.europa.eu/en/publications-data/shiga-toxinverocytotoxin-producing-escherichia-coli-stecvtec-infection-annual-0

 

Chapter on campylobacteriosis for 2017 

https://ecdc.europa.eu/en/publications-data/campylobacteriosis-annual-epidemiological-report-2017

 

Chapter on dengue for 2017 

https://ecdc.europa.eu/en/publications-data/dengue-annual-epidemiological-report-2017

 

Chapter on pertussis for 2017 

https://ecdc.europa.eu/en/publications-data/pertussis-annual-epidemiological-report-2017

 

Chapter on *Haemophilus influenzae* for 2017 

https://ecdc.europa.eu/en/publications-data/haemophilus-influenzae-annual-epidemiological-report-2017

 

Chapter on invasive meningococcal disease (IMD) for 2017 

https://ecdc.europa.eu/en/publications-data/invasive-meningococcal-disease-annual-epidemiological-report-2017

 

Chapter on gonorrhoea for 2017 

https://ecdc.europa.eu/en/publications-data/gonorrhoea-annual-epidemiological-report-2017

 

**ECDC communicable disease threats report**

Date of publication: every Friday

https://ecdc.europa.eu/en/threats-and-outbreaks/reports-and-data/weekly-threats

**

**Scientific peer-reviewed publications**

Publications by ECDC staff in scientific journals

Updated monthly

https://ecdc.europa.eu/en/publications-data?f%5b0%5d=output_types%3A1247

